# Malignancy in giant cell tumor of bone: analysis of an open-label phase 2 study of denosumab

**DOI:** 10.1186/s12885-020-07739-8

**Published:** 2021-01-22

**Authors:** Emanuela Palmerini, Leanne L. Seeger, Marco Gambarotti, Alberto Righi, Peter Reichardt, Susan Bukata, Jean-Yves Blay, Tian Dai, Danielle Jandial, Piero Picci

**Affiliations:** 1 Chemotherapy Unit, IRCCS Istituto Ortopedico Rizzoli, Department of Experimental, Diagnostic and Specialty Medicine (DIMES), Bologna University, 40136 Bologna, Italy; 2 David Geffen School of Medicine, UCLA Health System, 200 UCLA Medical Plaza Suite 165-57, Los Angeles, CA 90095 USA; 3 Department of Pathology, IRCCS Istituto Ortopedico Rizzoli, Bologna, Italy; 4 Department of Interdisciplinary Oncology, Sarcoma Center Berlin-Brandenburg; HELIOS Klinikum Berlin-Buch, Schwanebecker Chaussee 50, 13125 Berlin, Germany; 5 Department of Medical Oncology, Leon Berard Center, 28, rue Laennec 2 69373 Lyon Cedex 08, Lyon, France; 6 Global Development (Oncology), Amgen Inc., One Amgen Center Drive, MS 38-2-B, Thousand Oaks, CA 91320-1799 USA

**Keywords:** Denosumab, Giant cell tumor of bone, Bone neoplasms, RANK ligand

## Abstract

**Background:**

Giant cell tumor of bone (GCTB) is a rare osteoclastogenic stromal tumor. GCTB can rarely undergo malignant transformation. This post hoc analysis evaluated and classified malignancies in patients with GCTB who received denosumab.

**Methods:**

This analysis was conducted on patients with pathologically confirmed GCTB and measurable active disease treated with denosumab 120 mg subcutaneously once every 4 weeks, with loading doses on study days 8 and 15, as part of a phase 2, open-label, multicenter study. We identified potential cases of malignancy related to GCTB through an independent multidisciplinary review or medical history, associated imaging or histopathologic reports, and disease course. The findings were summarized and no statistical analysis was performed.

**Results:**

Twenty of five hundred twenty-six patients (3.8%) who received at least one dose of denosumab were misdiagnosed with GCTB that was later discovered to be malignancies: five primary malignant GCTB, five secondary malignant GCTB, four sarcomatous transformations, and six patients with other malignancies (giant cell-rich osteosarcoma, undifferentiated pleomorphic sarcoma, spindle cell sarcoma, osteogenic sarcoma, phosphaturic mesenchymal tumor of mixed connective tissue type, and fibrosarcoma/malignant fibrous histiocytoma). Many malignancies were present before denosumab was initiated (8 definitive cases, 7 likely cases), excluding potential involvement of denosumab in these cases. Signs associated with potential misdiagnoses of GCTB included poor mineralization with denosumab treatment, rapid relapse in pain, or a failure of the typical dramatic improvement in pain normally observed with denosumab.

**Conclusions:**

Although rare, GCTB can undergo malignant transformation, and rates in this study were consistent with previous reports. Signs of poor mineralization or lack of response to denosumab treatment may warrant close monitoring.

**Trial registration:**

clinicaltrials.gov, (NCT00680992). Registered May 20, 2008.

**Supplementary Information:**

The online version contains supplementary material available at 10.1186/s12885-020-07739-8.

## Background

Giant cell tumor of bone (GCTB) is a rare, osteoclastogenic stromal tumor [1]. GCTB is classified as an intermediate, locally aggressive, and rarely metastasizing tumor [2–4], with occasional distant slow-growing metastases. Furthermore, GCTB can undergo malignant transformation to high-grade sarcoma, such as osteosarcoma or undifferentiated sarcoma [4]. Malignancy in GCTB is a known risk reported to occur in 1 to 4% of patients and has been categorized as primary malignant (PM) GCTB, secondary malignant (SM) GCTB, or sarcomatous transformation [5]. PMGCTB is observed at initial GCTB diagnosis as an area of highly pleomorphic mononuclear cells coexistent and adjacent to an area of otherwise conventional benign GCTB area within the same lesion; therefore, sampling error in biopsies can be associated with missed diagnosis of PMGCTB [6]. Differential diagnoses of PMGCTB include giant cell-rich osteosarcoma, benign GCTB, aneurysmal bone cysts, chondroclastomas, and brown tumor of bone [7]. SMGCTB occurs at the site of a previously treated benign GCTB lesion and the pre-existing GCTB may be evident. SMGCTB is most commonly associated with prior radiation therapy [8], and is best considered as post-radiation sarcoma. However, SMGCTB may also occur after exclusive surgical treatment in the absence of prior radiotherapy [9]. Such cases are described as sarcomatous transformation of a previously documented benign GCTB. Misdiagnosis of GCTB, instead of PMGCTB or SMGCTB, can lead to improper treatment of an aggressive disease with typically poor prognosis [7].

Denosumab is a fully human RANKL inhibitor approved for use in adults and skeletally mature adolescents with GCTB that is unresectable or when surgical resection is likely to result in severe morbidity [10]. The purpose of this analysis was to evaluate and classify malignancies in patients with GCTB who were treated with denosumab as part of an open-label, phase 2 study [11].

## Methods

### Study design and patient population

The design, methods and results of the primary analysis of this phase 2, international, multicenter, open-label study (NCT00680992) have been published [11]. Briefly, eligible patients were adults and skeletally mature adolescents at least 12 years of age with pathologically confirmed GCTB and measurable active disease within 1 year of enrollment. Patients had Eastern Cooperative Oncology Group Performance Status score ≤ 2, no current use of alternative GCTB therapies, or known or suspected diagnosis of sarcoma, non-GCTB giant cell–rich tumors, brown cell tumor of bone, or Paget disease. Patients received 120 mg denosumab subcutaneously once every 4 weeks, with loading doses on study days 8 and 15. The study was approved by each site’s independent ethics committee (Additional file 1), and all adult patients provided written informed consent. For adolescents, written informed consent was required to be provided by the patient’s parent or legal representative, and assent of the adolescent obtained if requested by the ethics committee.

### Assessments

The purpose of this analysis was to examine potential cases of malignancy in GCTB. Potential malignancy cases were identified with an independent multidisciplinary review of all adverse events using the search term “neoplasms benign, malignant and unspecified (including cysts and polyps)”, which were then manually reviewed for clinical confirmation. We reviewed medical history, associated imaging or histopathologic reports, and disease course for all patients who met the search criteria, as well as those who discontinued study due to disease progression. We conducted an independent multidisciplinary review of trial reports of malignancy in GCTB using a panel of seven experts with extensive experience in bone sarcomas and GCTB (pathologist, radiologist, and medical and surgical oncologists were included). De-identified pathology, imaging, and medical history were systematically collected for review. Sites were requested to provide pathology and imaging samples, if available, from three time points: initial GCTB diagnosis, pre-enrollment biopsy, and malignant diagnosis. When pathology samples or imaging was not available, records were supplemented with the local transcribed imaging and pathologic reports.

The pathologist reviewed all submitted specimens (imaging was also provided); whenever possible, additional immunohistochemical stains and molecular testing were performed (see Additional file 2, Additional file 3). Each case was then reviewed in detail, including GCTB disease history, disease chronology including prior recurrences and therapies, timing of denosumab treatment, and occurrence of malignancy. The pathologist and radiologist presented their key findings, selected representative pathologic and radiographic key images and opinions to the panel, and consensus opinions were made for each patient.

Cases were classified as PMGCTB if review showed that malignancy was present at the time of diagnosis; SMGCTB was generally classified as malignancy occurring at the site of a previously treated benign GCTB lesion, typically, but not exclusively occurring after radiotherapy. Sarcomatous transformation was reserved for cases that could have truly resulted from potential exposure to denosumab. Patient numbers and age ranges, instead of age at treatment, were assigned for the purposes of this publication only and do not link to patients. The findings from the expert panel were summarized; no statistical analysis was performed.

## Results

### Patients

**Fig. 1:**
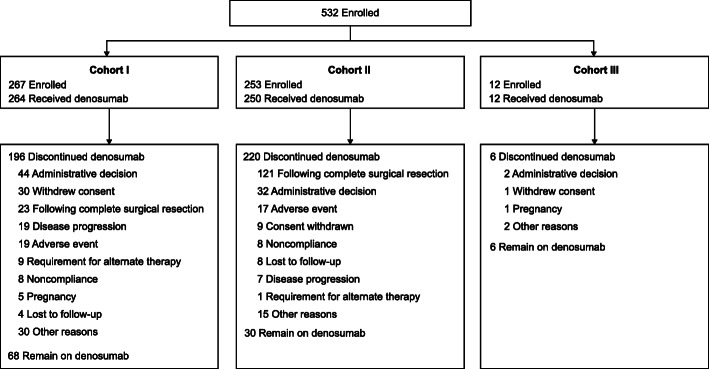
Profile of the randomized controlled trial. Patients were divided into three cohorts: patients with surgically unsalvageable tumors (Cohort I), patients with surgically salvageable tumors (Cohort II), and patients rolled over from a previous study (Cohort III)

A total of 532 patients (including 28 adolescents) were enrolled in the treatment phase (Fig. 1) and 526 patients received at least one dose of denosumab.

Patients identified with malignancies had baseline characteristics similar to the full study population, but were older (Table 1); the median (range) age was 52 (21–82) years in this analysis and 33 (13–83) years in the full study.

**Table 1:** Baseline demographics and characteristics^a^

	All patients (*N* = 532)	Malignancy patients (*n* = 20)
Women	301 (57)	12 (60)
Adolescents	28 (5)	0
Age, median (Q1, Q3), y	33 (25, 45)	52 (32, 65)
Follow-up, median (Q1, Q3), mo	58 (34, 74)	48 (17, 81)
No. of denosumab doses received, median (Q1, Q3)	34 (18, 61)	22 (11, 40)
GCTB disease type		
Primary resectable	168 (32)	6 (30)
Primary unresectable	94 (18)	2 (10)
Recurrent resectable	85 (16)	2 (10)
Recurrent unresectable	185 (35)	10 (50)
Prior GCTB surgery		
Yes	276 (52)	12 (60)
No	256 (48)	8 (40)
Prior GCTB radiotherapy		
Yes	52 (10)	4 (20)
No	480 (90)	16 (80)

*GCTB* giant cell tumor of bone, *mo* months, *y* years

^a^Data are n (%) unless indicated otherwise

**Fig. 2:**
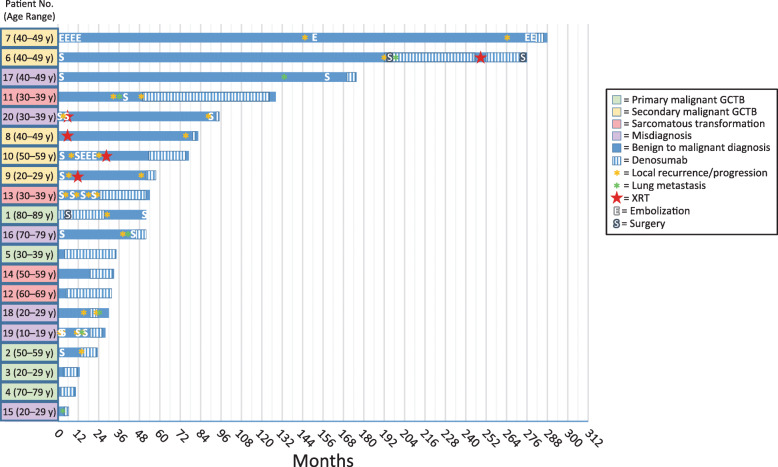
Summary of clinical courses of patients who developed malignancies during the primary study. Each bar represents one patient (patient number, age range in years); the length of the bar shows the length of time from benign diagnosis to malignant diagnosis. Patient numbers and age ranges (in brackets), instead of age at treatment, are identifiers for the purposes of this publication only and do not link to patients.  GCTB giant cell tumor of bone, XRT, radiation therapy

There were 20 cases of malignancy during the study: five PMGCTB cases, five SMGCTB cases, four sarcomatous transformations, and six other misdiagnoses. Malignancy cases included femur (*n* = 8), sacrum (*n* = 3), tibia (*n* = 2), pelvis, metatarsal, tibia, pubic ramus, lung, lung and navicular or cuneiform bones of foot, and humerus (*n* = 1 for each). A summary of the clinical courses of patients who developed malignancies during the primary study is presented in Fig. 2.

### Immunohistochemistry

Most patients (15/20, 75%) had some immunohistochemistry results (Table 2). Of the seven patients with both pre- and post-denosumab immunohistochemistry results, changes were observed in MDM2 expression (increased in six patients, decreased in one patient, unchanged in one patient), P53 (increased nuclear positivity in mononuclear cells for three patients, unchanged for three patients, decreased for one); P63 (decreased nuclear positivity in mononuclear cells in two, increased in one, unchanged in four patients). Six patients had *H3F3A* results: four were positive pre-denosumab and post-denosumab, and  two were positive pre-denosumab but had no post-denosumab measurement, and two were negative pre-denosumab but with no post-denosumab measurement (one PMGCTB and one misdiagnosis). Regarding pre-treatment samples, 12 cases were available for pathological revision, consisting of four biopsies and eight whole sections relative to curettages or emiresections.osteogenic sarcoma and fibroblastic

**Table 2:** Immunohistochemistry

Patient No.^**a**^ (Age Range)	Histology	Immunohistochemistry		H3F3A results
		Pre-denosumab	Post-denosumab	
**Primary GCTB**				
1 (80–89 y)	Undifferentiated pleomorphic sarcoma	NA	NA	NA
2 (50–59 y)	Undifferentiated spindle cell sarcoma	P63^+^, P53^–^, MDM2^+/–^ (FISH ^not amplified^)	P63^–^, P53^−^, MDM2^+^ (FISH ^not amplified^)	Pre-denosumab: H3F3A^–^ Post-denosumab: NE
3 (20–29 y)	Osteogenic sarcoma	KI67/MIB-1 40%, focally high, PDGFR-β^+^, PDGFR-α^–^	NA	NA
4 (70–79 y)	Undifferentiated pleomorphic sarcoma	NA	MDM2^–^ (FISH ^not available^), P53^–^, P63^–^, SATB2^−^	NE
5 (30–39 y)	Undifferentiated pleomorphic sarcoma	MDM2^+/–^ (FISH ^not available^) P53^–^, P63^−^	MDM2^+^(FISH ^not available^), P53^+^, P63^–^	NE
**Secondary GCTB**				
6 (40–49 y)	High-grade sarcoma	NA	NA	NA
7 (40–49 y)	Undifferentiated pleomorphic sarcoma	Vimentin^+^, P63^+^, CD31^–^, CD34^–^, CKCAM5.2^–^, AE1^–^/AE3^–^, SMA^–^, S100^–^, and desmin^–^	NA	NA
8 (40–49 y)	Undifferentiated pleomorphic sarcoma	NA	MDM2^+/–^ (FISH ^not available^), P53^+/–^, P63^−^	NA
9 (20–29 y)	Giant cell tumor with suspect progression to sarcoma	MDM2^+/–^ (FISH ^not available^), P53^+/–^, P63^+/–^, SATB2^+/–^	NA	Pre-denosumab: H3F3A^+^
10 (50–59 y)	High-grade undifferentiated spindle cell sarcoma	MDM2^+^ (FISH ^not available^), P53^–^, P63^+/–^, SATB2^+/–^ In recurrences: MDM2^–^ (FISH ^not available^), P53^–^, P63^–^, SATB2^+/–^	MDM2^+/–^ (FISH ^not available^), P53^+^, P63^+/–^, SATB2^–^	Pre-denosumab: H3F3A^+^ (at initial diagnosis and recurrences)
**Sarcomatous transformation**				
11 (30–39 y)	Undifferentiated spindle cell sarcoma	P63^–^, P53^+/–^, MDM2^–^ (FISH ^not available^);	P63^–^, P53^+^, MDM2^+^ (FISH ^not available^)	NA
12 (60–69 y)	High-grade osteosarcoma	P63^+^, P53^+^, MDM2^+^ (FISH ^not available^)	P63^–^, P53^+^, MDM2^+^ (FISH ^not available^)	Pre-denosumab: H3F3A^+^ Post-denosumab: H3F3A^+^
13 (30–39 y)	Undifferentiated spindle cell sarcoma	P53^+^, MDM2^+^ (by FISH ^amplified^) at malignant diagnosis but MDM2^–^ (FISH ^not amplified^) at initial GCTB diagnosis		NA
14 (50–59 y)	High-grade osteosarcoma	MDM2^+/–^ (FISH ^not available^), P53^+/–^, P63^−^	MDM2^+^ (FISH ^not available^), P53^–^, P63^+/–^, SATB2^+^	Pre-denosumab: H3F3A^+^
**Misdiagnoses**				
15 (20–29 y)	Giant cell-rich osteosarcoma	NA	SMA^+/–^, S100^–^, Ki67/MIB1 20%CD68^+^, vimentin^+^, focally positive for CD45 and SMA, S100^–^, CD30^–^, CD15^–^, Ki67 showed moderately high proliferative index	NA
16 (70–79 y)	Pleomorphic rhabdomyosarcoma	P63^–^, P53^+^, MDM2^+/–^ (FISH ^not available^)	P63^–^, P53^+^, MDM2^+^ (by FISH ^not amplified^), desmin^+^, myogenin^+^	Pre-denosumab: H3F3A^–^
17 (40–49 y)	Undifferentiated spindle cell sarcoma	CK AE1/AE3CD68^+^, vimentin^++^, cytokeratin AE 1/3^+^, S100^–^		NA
18 (20–29 y)	Osteogenic sarcoma (present pre-enrollment)	NA	NA	NA
19 (10–19 y)	Phosphaturic mesenchymal tumor of mixed connective tissue type	NA	NA	NA
20 (30–39 y)	Undifferentiated spindle cell sarcoma	NA	NA	NA

*CK* cytokeratin, *FISH* fluorescence in situ hybridization, *GCTB* giant cell tumor of bone, *MDM2* mouse double minute 2, *NA* not available, *NE* not evaluable, *PDGFR* platelet-derived growth factor receptors, *SATB2* special AT-rich sequence-binding protein 2, *SMA* smooth muscle antibody

^a^Patient numbers and age ranges (in brackets), instead of age at treatment, are identifiers for the purposes of this publication only and do not link to patients

FISH analysis for *MDM2* gene amplification was feasible in five samples; one case showed *MDM2* amplification. Most of the non-informative samples were characterized by poor fixation or excessive decalcification. A weak fluorescence signal in two other samples was found in tissue of poor quality (very old tissue blocks).

**Fig. 3:**
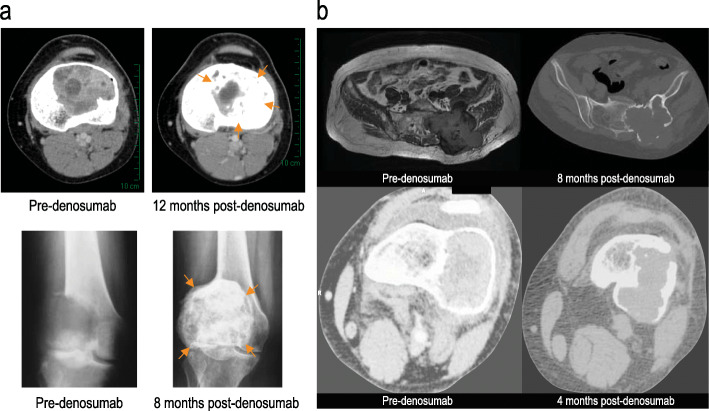
Misdiagnosis of GCTB. **a** Typical response of GCTB to denosumab leads to bone formation and calcification (top two images are axial CT soft tissue window and bottom two images are anteroposterior radiographs). **b** In misdiagnosed PMGCTB, poor calcification in response to denosumab is shown (top two images are axial T1-weighted MRI, axial CT bone windows and bottom two are axial CT soft tissue windows)

Typical responses of GCTB to denosumab treatment lead to bone formation or mineralization (Fig. 3). Six of nine (67%) patients with imaging for expert review showed decreased mineralization, which would otherwise be expected in response to denosumab: three PMGCTB, two sarcomatous transformations, and one misdiagnosis.

### PMGCTB

**Fig. 4:**
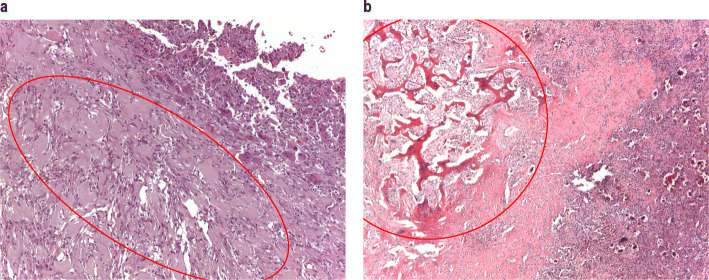
Histologic features of malignancy in GCTB. **a** Primary malignant GCTB, pre-denosumab: proliferation of ovoid to spindle bland-appearing cells, with scattered reactive multinucleated osteoclast-like giant cells *(top right of image)*, consistent with GCTB, juxtaposed to a proliferation of atypical spindle and pleomorphic cells, growing in fascicles, consistent with undifferentiated pleomorphic sarcoma *(red circle)*. **b** Secondary malignant GCTB, pre-denosumab, (recurrence in 2008): histological features consistent with GCTB *(bottom right of image)*, juxtaposed to a proliferation of atypical spindle cells, infiltrating in between the host bony trabeculae, consistent with high-grade undifferentiated spindle cell sarcoma *(red circle)*

PMGCTB was determined for five patients, three of which showed a pattern of imaging demonstrating poor mineralization. The malignant components in primary GCTB were undifferentiated pleomorphic sarcoma (*n* = 3), osteogenic sarcoma and fibroblastic osteosarcoma (*n* = 1 each) (Table 3). Durations of denosumab treatment ranged from 6 months to 29 months. The latency from initial diagnosis to PMGCTB diagnosis ranged from 10 months to 51 months. One patient (Patient 2) had a pathologic fracture at presentation. PMGCTB cases were characterized by a short latency period together with observations of poor responses to denosumab either on imaging or pain control, with progressive and aggressive clinical courses. For most of these patients, review of available baseline histology confirmed the presence of PMGCTB before enrollment and start of denosumab treatment (Fig. 4a).

**Table 3:** Malignancies in giant tumor of bone

Patient no.^a^ (age range)	Cohort	Site	Malignant histology	Radiation received, dose	Latency period	Duration of denosumab	Malignancy present prior to denosumab	Malignancy outcome
**Primary malignancies**								
1 (80–89 y)	Primary resectable	Femur	Initial diagnosis: appearance suspicious of sarcoma based on imaging atypical for GCTB and no lytic bone lesions Diagnosis: Undifferentiated pleomorphic sarcoma	No	4 y, 3 mo	2 y, 5 mo	Likely^b^	Death after postsurgical complications from femur replacement
2 (50–59 y)	Recurrent unresectable	Femur	Giant cell tumor with pleiomorphic spindle cells, suspect malignant areas, extensive reactive changes due to fracture Diagnosis: Undifferentiated spindle cell sarcoma	No	2 y	10 mo	Likely^b^	Chemotherapy with complete response
3 (20–29 y)	Primary resectable	Pelvis	Numerous cores, largest was 1.5 cm, consisting of brown, friable tissue; GCTB with atypical features and high expression of proliferation markers Diagnosis: Osteogenic sarcoma	No	13 mo	6 mo	Likely^b^	Lost to follow-up
4 (70–79 y)	Primary resectable	Sacrum	Spindle cell proliferation and cellular atypia, no giant cells. Suggests malignant transformation characterized by aspects of pleomorphism and cellular atypia with mitotic index elevated Diagnosis: Undifferentiated pleomorphic sarcoma	No	10 mo	8 mo	Yes	Death of primary disease 3 mo after malignancy diagnosis
5 (30–39 y)	Primary resectable	Tibia	Malignant spindle cell sarcoma (no maturation due to denosumab) Diagnosis: Undifferentiated pleomorphic sarcoma	No	2 y, 8 mo	2 y, 4 mo	Yes	Patient underwent planned amputation plus chemotherapy
**SMGCTB**								
6 (40–49 y)	Recurrent unresectable	Metatarsus	Diagnosis: High-grade sarcoma	Yes, 50 Gy	13 y	6 y, 3 mo	No	Amputation plus chemotherapy; deceased 12 mo after malignancy diagnosis
7 (40–49 y)	Recurrent unresectable	Sacrum	At enrollment, lesion contained few giant cells. In foci, sheets of cells exhibiting epithelioid morphology with cytologic atypical and brisk mitotic activity, including atypical mitoses Diagnosis: Undifferentiated pleomorphic sarcoma	No	25 y	4 mo	Likely^b^	Deceased 6 mo after malignancy diagnosis
8 (40–49 y)	Recurrent resectable	Tibia	Malignant spindle cell sarcoma (no maturation due to denosumab) Diagnosis: Undifferentiated pleomorphic sarcoma	Yes, 56 Gy	7 y, 8 mo	1 mo	Yes	Amputation plus chemotherapy; alive at last follow-up
9 (20–29 y)	Recurrent resectable	Femur	Areas of solid ABC and GCTB associated with epithelial and spindle cell proliferation. Diagnosis: GCTB with suspect progression to sarcoma	Yes, 56 Gy	4 y, 8 mo	6 mo	Likely^b^	Lung metastases diagnosed soon after femur malignancy; deceased 5 mo after malignancy diagnosis
10 (50–59 y)	Recurrent unresectable	Sacrum	Diagnosis: High grade undifferentiated spindle cell sarcoma, consistent with differentiation arising in malignant GCTB	Yes, 25 fractions	6 y, 7 mo	1 y, 8 mo	Yes	Surgery (resection and curettage, laminectomy); deceased 2 mo after malignancy diagnosis
**Sarcomatous transformation**								
11 (30–39 y)	Recurrent unresectable	Distal femur	Diagnosis: Undifferentiated spindle cell sarcoma	No	11 y	6 y, 2 mo	No	Amputation and chemotherapy; alive at last follow-up
12 (60–69 y)	Primary resectable	Tibia	Diagnosis: High-grade osteosarcoma	No	2 y, 11 mo	2 y, 2 mo	No	Chemotherapy and amputation; deceased 7 mo after malignancy diagnosis
13 (30–39 y)	Recurrent resectable	Distal femur	Diagnosis: Undifferentiated spindle cell sarcoma	No	4 y, 9 mo	2 y, 5.5 mo	No	Tumor resection and prosthesis plus chemotherapy; alive, no evidence of disease at last follow-up
14 (50–59 y)	Primary resectable	Distal femur	Microscopic picture corresponds to chondroblastic/osteoblastic osteosarcoma high grade that probably developed from GCTB Diagnosis: High-grade osteosarcoma	No	1 y, 5 mo	1 y, 2 mo; progression after only 3 mo of treatment	No	Amputation plus chemotherapy; alive at last follow-up

*ABC* aneurysmal bone cyst, *GCTB* giant cell tumor of bone, *Gy* Gray unit, *mo* months, *SMGCTB* secondary malignant giant cell tumor of bone, *y* years

^a^Patient numbers and age ranges (in brackets), instead of age at treatment, are identifiers for the purposes of this publication only and do not link to patients

^b^Malignancy likely, but not definitively, present prior to denosumab due to lack of sufficient biopsies for expert review; opinion of expert reviewers based on available evidence (existing biopsy samples or local pathologist report) as noted in the “malignant histology” column)

### SMGCTB

Five of 20 patients with malignancy developed SMGCTB, four after previous radiotherapy (Table 3). Compared with PMGCTB, patients with SMGCTB had longer latency from initial GCTB diagnosis, ranging from 56 months up to 25 years. Duration of denosumab treatment ranged from 1 month to 75 months. For four patients, malignancy was definitively present (Patients 8 and 10) or likely present before denosumab treatment, as verified by re-examination of pathology samples or report (Fig. 4b). A malignancy was classified as likely, but not definitively present, before denosumab, when there was a lack of sufficient biopsies for expert review (Patients 7 and 9). Patient 6 developed a malignancy after radiotherapy and denosumab treatment, but did not have pre-enrollment pathology available for review.

### Sarcomatous transformation of GCTB

Four patients had malignancy deemed as true sarcomatous transformation (ie, not previously treated for GCTB) (Table 3). Patients with sarcomatous transformation presented with GCTB in the femur or tibia. Two patients had pathologic fracture at presentation (Patients 11 and 12). Unlike many cases of primary or SMGCTB, sarcomatous transformation was not present before receiving denosumab for all four patients. The time from diagnosis of GCTB to diagnosis of sarcomatous transformation ranged from 17 months to 11 years. Patients received denosumab from 14 months to 6 years, with the time from initial GCTB diagnosis to initiation of denosumab ranging from 2 months to 4 years.

### Malignancies misdiagnosed as benign GCTB

A summary of the six patients determined to have misdiagnosis of benign GCTB is presented in Table 4. Two of these patients had lung nodules as the primary target lesion at the time of study enrollment (Patients 17 and 19); most patients were in the recurrent unresectable cohort. After re-examination, the correct diagnoses were undifferentiated spindle cell sarcoma (2 patients) and giant cell-rich osteosarcoma, pleomorphic rhabdomyosarcoma, osteogenic sarcoma, and phosphaturic mesenchymal tumor of mixed connective tissue type (1 patient each). Of note, two of these rare diagnoses included second pathologic opinions by global experts outside of the panel. Four patients were pathologically confirmed to have the malignancy present before denosumab treatment (Patients 17–20); for the other two (Patients 15 and 16), the malignancy was classified as likely, but not definitively, present before denosumab, based on imaging and clinical history, but it was not possible to confirm pathologically given that there were no available specimens or lack of adequate specimens for pathologic re-evaluation.

**Table 4:** Misdiagnosis of benign GCTB

Patient no.^a^ (age range)	Cohort	Site at enrollment	Malignant histology	Malignancy present prior to denosumab?	Malignancy outcome
15 (20–29 y)	Primary resectable	Distal femur	Giant cell-rich osteosarcoma	Likely^b^	Amputation; deceased 12 mo after malignancy diagnosis
16 (70–79 y)	Recurrent unresectable	Pubic ramus	Pleomorphic rhabdomyosarcoma	Likely^b^	Deceased 6 mo after malignancy diagnosis
17 (40–49 y)	Recurrent unresectable	Lung	Undifferentiated spindle cell sarcoma	Yes	Deceased 4 mo after malignancy diagnosis
18 (20–29 y)	Recurrent unresectable	Femur	Osteogenic sarcoma (present pre-enrollment)	Yes	Chemotherapy and resection of tumor; alive at last follow-up
19 (10–19 y)	Recurrent unresectable	Lung and cuneiform bones of foot	Phosphaturic mesenchymal tumor of mixed connective tissue type	Yes	Alive at last follow-up with progressive disease
20 (30–39 y)	Recurrent resectable	Humerus	Undifferentiated spindle cell sarcoma	Yes	Alive at last follow-up

*GCTB* giant cell tumor of bone, *mo* months,* y* years

^a^Patient numbers and age ranges (in brackets), instead of age at treatment, are identifiers for the purposes of this publication only and do not link to patients

^b^Malignancy likely, but not definitively, present prior to denosumab due to lack of sufficient biopsies for expert review; opinion of expert reviewers based on available evidence

## Discussion

Patients rarely develop malignancy in GCTB. In this study, the overall incidence of an adverse event of new malignancy in GCTB was 3.8% (20/526): five (1.0%) had PMGCTB, five (1.0%) had SMGCTB, four (0.8%) had sarcomatous transformation, and six (1.0%) had a misdiagnosis of benign GCTB. This malignancy rate in GCTB during the study, including sarcomatous transformation, was not higher than historical rates (range 1–15%) [7, 8, 12–17]. A recent review of 2315 patients with GCTB in the pre-denosumab era indicated that the cumulative rate of malignancy was 4.0%; the cumulative incidences of primary and SMGCTB were 1.6 and 2.4%, respectively; these rates are comparable to the present study [5].

Historically, malignancies in GCTB are secondary malignancies typically observed after radiotherapy [8, 18], but they may also occur after surgical treatment, such as bone grafts, without adjuvant radiotherapy [9]. Notably, one patient categorized as having SMGCTB did not receive radiotherapy: this categorization was based on the fact that malignancy was present prior to denosumab therapy. Primary malignancies are considered to be rare [7]. There have been some observations of sarcoma development in patients with GCTB treated with denosumab in a phase 2 trial, none of which were thought to be caused by denosumab [11]. However, as concern has been expressed about the possible risk of malignant transformation associated with denosumab, the best approach is to assess this risk in the context of potential complications introduced by misdiagnosis [19]. Diagnosis of malignancy in GCTB can be challenging because radiologic features of primary malignancy are often identical to those of benign GCTB [6, 7]. Furthermore, there is a high level of heterogeneity among primary malignant tumors [6]. As a result, primary malignancies may only be observed after re-examination. We also found six misdiagnoses; this is not a surprising finding as these malignancies, along with GCTB, contain giant cells and are rare and therefore difficult to diagnose. The lack of clear diagnostic criteria for malignant GCTB further complicates diagnosis. From a pathological point of view, the main feature that represents a potential misdiagnosis is the presence of a giant cell component that can be present in other malignant mesenchymal tumors. Other signs of potential misdiagnosis of GCTB may include poor mineralization or rapid relapse in pain or no pain relief during treatment with denosumab. The use of comprehensive histologic sampling, careful follow-up, and timely treatment of local recurrence is therefore recommended [5].

In our study, 67% of the nine malignancies with evaluable imaging showed poor mineralization with denosumab treatment and in many cases were also accompanied by a rapid relapse in pain or a failure of the typical dramatic improvement in pain response that is usually observed with denosumab initiation [11]. Mineralization is an early indication of response and an expected finding of denosumab treatment based on its mechanism of action; therefore, in cases where denosumab treatment does not lead to adequate pain control or demonstrates less than anticipated tumor mineralization, clinical reassessment is suggested with consideration for potential misdiagnosis and re-biopsy of the lesion [20].

Currently, there are no well-established tumor response criteria for patients with GCTB [21]. Radiological classification based on computed tomography (CT) images may be more accurate than Choi criteria in identifying early tumor changes due to denosumab therapy [22]. Based on post hoc analysis of patients with GCTB treated with denosumab, increased positron-emission tomography avidity may accurately identify malignant changes [20]. Clinical presentation is important to distinguish patients without symptoms or pain, as well as those with increasing symptoms. Routine follow-up with CT with comparison to all prior imaging, including baseline studies, is essential. The interval between imaging should be left to physician’s discretion according to patient characteristics.

Difficulties exist in distinguishing normal post-denosumab bone histopathology changes from malignant changes. Histopathologic changes associated with denosumab in GCTB include a total or near-total disappearance of osteoclast-like giant cells; residual tumor cells are primarily normal-appearing spindle cells arranged in fascicles often with storiform pattern [23]. Osteoid production with variable degree of mineralization is typically present [23]. A case report of a patient with GCTB treated with nine cycles of denosumab showed pseudosarcomatous spindle cell proliferation with osteoid matrix resembling osteosarcoma [24]. Such observations after denosumab treatment further complicate proper diagnosis of secondary malignancies.

Only seven patients had both pre- and post-denosumab immunohistochemistry available. It is interesting to observe an increase in nuclear expression of P53 in secondary GCTB or sarcomatous transformation, which has been previously implicated in malignant transformation of GCTB [6, 25]. *H3F3A* encodes the replication-independent histone H3.3; 49 to 92% of patients with GCTB have mutations in *H3F3A* (typically G34W); chondroblastoma has been associated with mutations in *H3F3B*; other giant cell containing tumors had no or few mutations in either of these genes [26–28]. One patient with PMGCTB (Patient 2) and one with a misdiagnosis (Patient 16) who had undifferentiated pleomorphic sarcoma were negative for *H3F3A* mutations. Because of the high rate of *H3F3A* mutations in GCTB, patients negative for mutations should be suspected of having other bone tumor types and followed closely. One case (Patient 13) showed *MDM2* amplification in the malignant transformation but not in the initial GCTB diagnosis, suggesting a possible role of *MDM2* amplification in the development of malignancy.

Fifteen of the 20 malignancies were definitively or likely present before denosumab was initiated, which excludes any potential involvement of denosumab in these cases. Denosumab has established safety through several clinical studies in patients with GCTB and in patients with solid tumors used for prevention of skeletal-related events [29–31]. The RANK/RANKL pathway is also known to be involved in tumorigenesis [32, 33]. GCTB consists of stromal cells expressing RANKL and osteoclast-like giant cells expressing the RANK receptor, and signaling through the RANK receptor contributes to osteolysis and tumor growth [31, 34]. Denosumab blocks RANKL from binding to its receptor on the surface of osteoclasts, their precursors, and osteoclast-like giant cells [20]. Based on this, it is highly unlikely that denosumab would increase the risk of malignancy [35]. The incidence of malignancy was not higher than historical rates, which also supports no involvement of denosumab.

## Conclusions

In conclusion, we identified 20 malignancies in an open-label study, for an overall rate of 3.8%, which is not higher than historical rates. Out of those malignancies, six were misdiagnoses of benign GCTB. Furthermore, 15 out of 20 malignancies were definitively or likely present before denosumab was initiated, excluding any potential involvement of denosumab in these cases. Given the rarity of this complication in the already rare GCTB, careful diagnosis and follow-up are recommended.

## Supplementary Information

**Additional file 1.**

## Data Availability

Qualified researchers may request the dataset supporting the conclusions of this article from Amgen. Complete details are available at the following: https://www.amgen.com/science/clinical-trials/clinical-data-transparency-practices/clinical-trial-data-sharing-request/

## Abbreviations

CT: Computed tomography
GCTB: Giant cell tumor of bone
MDM2: Mouse double minute 2
PM: Primary malignant
PMGCTB: Primary malignant giant cell tumor of bone
SM: Secondary malignant
SMGCTB: Secondary malignant giant cell tumor of bone
