# Supplementary material for: Malignancy in giant cell tumor of bone: analysis of an open-label phase 2 study of denosumab
Source: BMC Cancer. 2021 Jan 22;21:89. doi: 10.1186/s12885-020-07739-8 (PMC7824947; doi:10.1186/s12885-020-07739-8)
Supplement: Supplementary file 1 — Additional file 1. [file 12885_2020_7739_MOESM1_ESM.pdf]

Supplementary Information

Malignancy in Giant Cell Tumor of Bone: Analysis of an Open-Label Phase 2 Study of Denosumab

Emanuela Palmerini, MD, PhD, Leanne L Seeger, MD, Marco Gambarotti, MD, Alberto Righi, MD, Peter Reichardt, MD, Susan Bukata, MD, Jean-Yves Blay, MD, Tian Dai, PhD, Danielle Jandial, MD, Piero Picci, MD

Additional file 1: List of Independent Ethics Committees (IECs)

Table S1. List of IECs

Investigator	Site name	Location	IEC name
Chawla, Sant	Sarcoma Oncology Center	Santa Monica CA, USA	Sarcoma Oncology Center Ethics Committee
Skubitz, Keith	University of Minnesota	Minneapolis MN, USA	University of Minnesota, Institutional Review Board
Schuetze, Scott	Michigan Medicine: University of Michigan	Ann Arbor, MI, USA	University of Michigan Hospital and Health Centers Ethics Committee
Hartner, Lee	Pennsylvania Hospital	Philadelphia, PA, USA	Hospital of the University of Pennsylvania Ethics Committee
Choy, Edwin	Massachusetts General Hospital	Boston, MA, USA	Executive Committee on Research
Henshaw, Robert	MedStar Washington Hospital Center	Washington DC, USA	Medstar Health Research Institute Institutional Review Board
Malamud, Stephen	Mount Sinai Heath System	New York, NY, USA	Mount Sinai Ethics Committee
Scarborough, Mark	University of Florida, College of Medicine	Gainesville, FL, USA	University of Florida, Institutional Review Board
Bryant, Nichole	Prisma Health Greenville Memorial Hospital	Greenville, SC, USA	University of South Carolina, School of Medicine, Ethics Committee
Ganjoo, Kristen	Stanford Health Care, Stanford University Medical Center	Stanford, CA, USA	Stanford Center for Biomedical Ethics
Casali, Paolo	Istituto Nazionale Tumori	Milano, Italy	INT Ethics Committee
Ferrari, Stefano	Istituto Ortopedico Rizzoli	Bologna, Italy	Central Emilia Wide Area Ethical Committee of the Emilia-Romagna Region
Blay, Jean Yves	Leon Berard Center	Lyon cedex 8, France	IARC Ethics Committee
Duffaud, Florence	Aix Marseille Université	Marseille cedex 05, France	Aix Marseille Université Ethics Committee
Le Cesne, Axel	Institut de Cancerologie Gustave-Roussy	Villejuif cedex, France	Department for Sponsorship of Clinical Studies
Hamilton, Anne	Peter MacCallum Cancer Centrre	East Melbourne, Victoria, Australia	Human Research Ethics & Governance: Peter Mac Ethics Committee
Powell, Alex	Hollywood Private Hospital	Nedlands, Western Australia, Australia	Hollywood Private Hospital Research Ethics Committee
Stalley, Paul	The University of Sydney	Camperdown, New South Wales, Australia	Human Research Ethics Committee
Rutkowski, Piotr	Maria Sklodowska-Curie Memorial Cancer Center and Institute of Oncology (MSCI)	Warszawa, Poland	MSCI Ethics Committee
Rychlowska-Pruszynska, Magdalena	Institute of Mother and Child	Warszawa, Poland	Institute of Mother and Child Bioethics Committee

Investigator	Site name	Location	IEC name
Pink, Daniel	Klinik für Hämatologie/Onkologie und Palliativmedizin, HELIOS Klinikum	Bad Saarow, Germany	Ethik-Kommission der Landesärztekammer Brandenburg
Gelderblom, Andre	Leiden University Medical Center	Leiden, Netherlands	ICLON Research Ethics Committee
Parry, Michael	Royal Orthopaedic Hospital	Birmingham, UK	Research Ethics Committee
Luna, Pablo	Hospital Son Espases	Palma de Mallorca, Baleares, Spain	Hospital Research Committee and the Institutional Ethical Review Board
Lopez Pousa, Antonio	Hospital de la Santa Creu i Sant Pau	Barcelona, Cataluña, Spain	Ethics Committee for research with medicinal products (CEIm)
Razak, Albiruni	Mount Sinai Hospital	Toronto, Ontario, Canada	Mount Sinai Hospital Research Ethics Board
Alcindor, Thierry	McGill University	Montreal, Quebec, Canada	Research Ethics Board
Engellau, Jacob	Lund University Hospital	Lund, Sweden	Swedish Ethical Review Authority
Funovics, Philipp	Medical University of Vienna	Wien, Austria	Ethics Committee of the Medical University of Vienna

Additional file 2: Supplementary Methods

Immunohistochemistry

The tissue was fixed in 4% buffered formalin, routinely processed and embedded in paraffin; 4 µm-thick tissue sections were cut, heated at 58°C for 2 hours, deparaffinized and immunostained on a Ventana BenchMark following the manufacturer's guidelines (Ventana Medical Systems, Tucson AZ, USA or Dako Autostaniner Plus, Dako Glostrup, Denmark). The reaction was revealed with iVIEW DAB detection kit, providing a brown reaction product. Table S2 shows antibody source, dilution and antigen retrieval protocol. Pretreatment for antigen retrieval was performed at 95°C with Tris-EDTA pH 8 for 20 minutes. Appropriate positive and negative controls were included in each run.

Fluorescence in situ hybridization (FISH)

FISH was performed using the SPEC MDM2/CEN 12 Dual Color Probe (ZytoVision GmbH, Bremerhaven, Germany) according to the manufacturer's protocol. Tissue sections of 4 µm were dewaxed in xylene and treated with an ethanol-to-water series. Sections were incubated in TRIS 5 mM-EDTA 1 mM solution at 95°C for 15 minutes, rinse in distilled water and treated with pepsin solution (0.04% in 0.01N HCl) at 37°C for 15 minutes. The MDM2-probe was applied to the target area and the slides were coverslipped and sealed with rubber cement. The samples and probe were co-denaturated in Dako Hybridizer (Dako, Glostrup, Denmark) at 85°C for 1 minute and incubated overnight at 37°C. Cases were scored by counting a minimum of 100 tumor cell nuclei at 100X magnification with a DAPI/green/red triple band pass filter. The number of MDM2 and CEP12 signals was determined and a MDM2/CEP12 ratio was calculated for each nucleus. A ratio >2.0 in at least 10% of nuclei was considered amplified for the MDM2 gene.

Additional file 3: Table S2

Source of Dilution and Antigen Retrieval of the Antibodies Used

Antibodies	Clone	Source	Dilution/Antigen retrieval
CK AE1/AE3	AE1/AE3/PCK26	monoclonal antibody	Prediluted/Ventana
Smooth muscle actin	1A4	monoclonal antibody	Prediluted/Ventana
Desmin	DE-R-11	monoclonal antibody	Prediluted/Ventana
Myogenin	EP162	monoclonal antibody	Pre-diluted/Ventana
S100	4C4.9	monoclonal antibody	Pre-diluted/Ventana
CD31	JC70	monoclonal antibody	Pre-diluted/Ventana
MDM2	IF2	mouse monoclonal antibody	1:50/ Invitrogen
CD34	QBEnd-10	monoclonal antibody	pre-diluted/Ventana
SATB2	SATBA4B10	mouse monoclonal antibody	1:200/ Abcam
H3F3A (G34W)	RM263	rabbit monoclonal antibody	1:600/Histoline
P53	DO-7	monoclonal antibody	Pre-diluted/Ventana
P63	4A4	mouse monoclonal antibody	Prediluted/Ventana
Ki67	30/9	monoclonal antibody	Pre-diluted/Ventana
